# P-581. Racial/Ethnic Disparities of Renal Outcomes in People with HIV (PWH) on Tenofovir Alafenamide (TAF) Based Antiretroviral Treatment (ART) Regimens in the Real World

**DOI:** 10.1093/ofid/ofae631.779

**Published:** 2025-01-29

**Authors:** Samir K Gupta, Robert Canada, Sarjita Naik, Xiwen Huang, Amy Weinberg, Lauren Temme, Li Tao, Betty Chiang, Anand Chokkalingam, Jihad Slim

**Affiliations:** Indiana University School of Medicine, Indianapolis, Indiana; University of Tennessee Health Science Center, Memphis, Tennessee; Gilead Sciences, Inc., Foster City, California; Gilead Sciences, Inc., Foster City, California; Gilead Sciences, Inc., Foster City, California; Gilead Sciences, Foster City, CA; Gilead Sciences, Foster City, CA; Gilead Sciences, Inc., Foster City, California; Gilead, Foster City, California; Saint Michael’s Medical Center, Newark, NJ, USA, Newark, New Jersey

## Abstract

**Background:**

The risk of chronic kidney disease (CKD) is higher in PWH than HIV-negative persons. Black communities are not only disproportionately affected by HIV but also more at risk for kidney disease than other races. Despite Black PWH generally being underrepresented in clinical trials, the BRAAVE trial showed switching to bictegravir/emtricitabine/TAF demonstrated non-inferior efficacy and similar tolerability, including renal safety, compared to prior ART regimens in this group. With this analysis, we aim to assess whether these trial results translate to the real-world setting among Black PWH using a retrospective cohort analysis.

**Figure:**
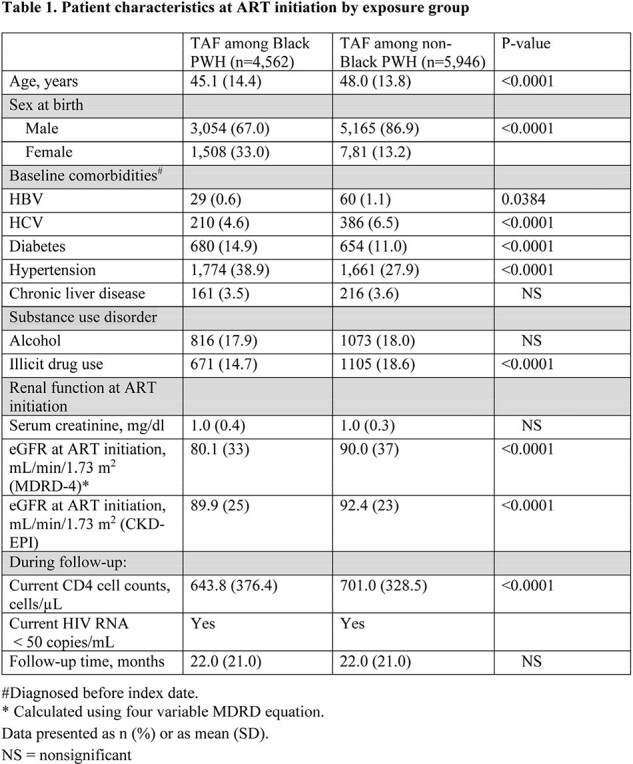

**Methods:**

The descriptive analysis was conducted using IQVIA ambulatory electronic medical record (EMR) database (November 2015 – July 2023). The study population included PWH (≥ 18 years) who were newly prescribed with ART regimens. Study endpoints evaluated the incidence of azotemia, Fanconi syndrome, renal tubular acidosis (RTA), renal toxicity, acute kidney injury (AKI), and CKD stages using diagnosis codes, stratified by racial group (Black and non-Black) and treatment with TAF-based regimens using prescription codes. Incidence rates were reported. The analysis was conducted using ATLAS Version 2.12.1.

**Figure:**
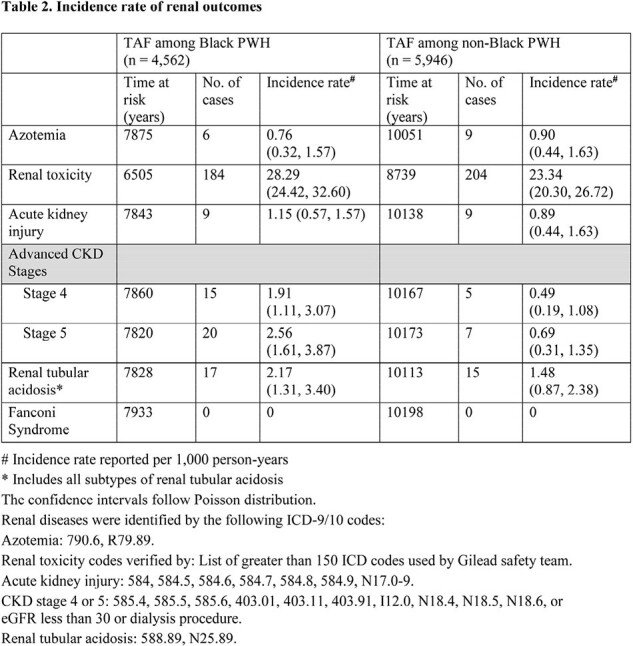

**Results:**

Overall, 4,562 Black and 5,946 non-Black PWH were identified on TAF-containing regimen, with 7,933 and 10,198 person-years of follow-up, respectively (Table 1). Compared to non-Black PWH, Black PWH were more likely to be female, have diabetes and hypertension. Black PWH on TAF had a higher incidence of advanced CKD and similar incidence rates of azotemia, renal toxicity, AKI, and RTA compared to non-Black PWH on TAF (Table 2). When stratified by baseline hypertension, no significant differences in the incidence of renal disease were observed between Black and non-Black PWH. There were no cases of Fanconi syndrome in the study cohort.

**Conclusion:**

Despite the inherent limitations of real-world data, this analysis included a large cohort of Black PWH on TAF-containing ART and showed a low incidence of RTA and AKI, despite predisposition to increased rates of renal decline or safety outcomes in Black PWH starting ART.

**Disclosures:**

**Samir K. Gupta, MD**, Gilead Sciences, Inc.: Advisor/Consultant|ViiV Healthcare: Advisor/Consultant|ViiV Healthcare: Grant/Research Support **Sarjita Naik, PharmD, MPH**, Gilead Sciences, Inc.: Employee|Gilead Sciences, Inc.: Stocks/Bonds (Private Company) **Xiwen Huang, PhD**, Gilead Sciences, Inc.: Employee|Gilead Sciences, Inc.: Stocks/Bonds (Public Company) **Amy Weinberg, DNP, MS**, Gilead Sciences, Inc.: Employee|Gilead Sciences, Inc.: Stocks/Bonds (Private Company) **Lauren Temme, PharmD**, Gilead Sciences, Inc.: Employee|Gilead Sciences, Inc.: Stocks/Bonds (Private Company) **Li Tao, MD, PhD**, Gilead Sciences, Inc.: Employee|Gilead Sciences, Inc.: Stocks/Bonds (Public Company) **Betty Chiang, MD**, Gilead Sciences, Inc.: Employee|Gilead Sciences, Inc.: Stocks/Bonds (Private Company) **Anand Chokkalingam, PhD**, Gilead Sciences, Inc.: Employee|Gilead Sciences, Inc.: Stocks/Bonds (Public Company) **Jihad Slim, MD, FACP**, AbbVie: Grant/Research Support|AbbVie: Honoraria|AbbVie: Speaker Bureau|Gilead Sciences, Inc.: Grant/Research Support|Gilead Sciences, Inc.: Honoraria|Gilead Sciences, Inc.: Speaker Bureau|Merck: Grant/Research Support|Merck: Honoraria|Merck: Speaker Bureau|Theratechnologies: Advisor/Consultant|Theratechnologies: Honoraria|ViiV Healthcare: Advisor/Consultant|ViiV Healthcare: Grant/Research Support|ViiV Healthcare: Speaker Bureau

